# Case report: Treatment of cyclobenzaprine ingestion in two dogs with intravenous intralipid therapy

**DOI:** 10.3389/fvets.2024.1354028

**Published:** 2024-02-12

**Authors:** Kaitlyn Dreese, Adesola Odunayo, Melissa C. Bucknoff

**Affiliations:** ^1^College of Veterinary Medicine, University of Florida, Gainesville, FL, United States; ^2^Ross University School of Veterinary Medicine, Basseterre, Saint Kitts and Nevis

**Keywords:** case report, cyclobenzaprine, intralipid emulsion, intralipid emulsion therapy, skeletal muscle relaxant

## Abstract

**Introduction:**

The objective of this case series is to describe the clinical signs and outcome of cyclobenzaprine ingestion in two dogs treated with intralipid emulsion (ILE) and supportive care.

**Case or series summary:**

Two dogs presented for evaluation of cyclobenzaprine ingestion. A 4-year-old female spayed Rat Terrier (dog 1) presented within 4 h of ingestion of cyclobenzaprine (between 9.7 and 25.9 mg/kg). The dog experienced abnormal behavior, agitation, tremors, tachycardia, and hypertension. There were no significant clinicopathological abnormalities. The dog was treated with ILE, cyproheptadine, and activated charcoal. All clinical signs resolved after treatment. A 5-month-old female intact mixed-breed dog (dog 2) presented after ingestion of an unknown amount of cyclobenzaprine 2–3 h prior to presentation. The dog experienced dull mentation, tremors, loss of gag reflex, tachycardia, and hypertension. There were no significant clinicopathological abnormalities. Orogastric decontamination was performed via gastric lavage, and activated charcoal was given via orogastric tube, followed by ILE. All clinical signs resolved after therapeutic intervention.

**Discussion:**

This is the first report documenting clinical signs of cyclobenzaprine toxicity in two dogs followed by successful treatment with gastric emptying, ILE, and supportive care.

## Introduction

Cyclobenzaprine is a skeletal muscle relaxant prescribed in humans for the treatment of muscle spasms, pain, and fibromyalgia (1–11). The mechanism of action of cyclobenzaprine is largely unknown but is suspected to work at the level of the brainstem within the central nervous system (CNS) without interfering with skeletal muscle function (2–4, 6, 8, 9, 12–14). It is thought to target serotonin, norepinephrine, dopamine, and Toll-like receptors (4, 6, 8, 11, 15). Skeletal muscle relaxation occurs via inhibition of efferent alpha and gamma motor neurons (4, 6, 13).

Cyclobenzaprine was initially investigated as an anti-depressant given its similarity to tri-cyclic anti-depressants (TCAs) (3–6, 9, 11, 15), only differing in chemical structure by a single double bond (3, 5, 6, 15). It is due to these similarities to TCAs that cyclobenzaprine overdose was initially concerning for life-threatening cardiovascular and neurological adverse effects. However, in a 5-year retrospective analysis of cyclobenzaprine overdose in human patients, there were no reported deaths from dysrhythmias or seizures, though 13 out of 755 cases required mechanical ventilation (3). In that study, the highest reported dose was 1 g (12.5 mg/kg based on a standard 80-kg adult), and the mean onset of clinical signs was within 1.4 h (3). Signs of toxicity have been reported in humans after ingestion of 100 mg (1.25 mg/kg based on a standard 80-kg adult) (4).

The use of cyclobenzaprine reported in the literature in dogs is very limited. Two studies evaluated oral bioavailability and excretion of cyclobenzaprine in dogs. The first study administered a dose of 30 mg/kg by mouth once a day for 3 days to Beagle dogs (2). Neither study evaluated for nor reported complications associated with cyclobenzaprine administration. To the authors’ knowledge, there is no publication in the literature describing accidental cyclobenzaprine ingestion and toxicity in dogs.

Common clinical signs in humans of cyclobenzaprine overdose include lethargy, dizziness, confusion, disorientation, agitation, psychosis, tachycardia, dysrhythmias, hypertension, hypotension (although uncommon), mydriasis, urinary retention, tremors, myoclonus, seizures, and coma (3, 4, 6–8, 11, 16). Renal insufficiency along with rhabdomyolysis was reported in a patient with prolonged agitation (4). In severe cases, respiratory failure that required mechanical ventilation was found to occur (4).

In humans, the oral bioavailability of cyclobenzaprine is between 33 and 55% (9, 13, 14) and peak plasma levels may not be reached for 3 to 8 h after ingestion (11, 12). Hucker et al. (1) showed that peak plasma levels in dogs are reached after 2 h when given a 2 mg/kg dose orally or intravenously. Cyclobenzaprine is 93% protein-bound (9, 11, 17), is widely distributed to the tissues (14, 17), and undergoes enterohepatic recirculation (12–14). It is extensively metabolized in the liver by cytochrome P450, 1A2, and 3A4 (9, 11, 13–15). While some metabolites are excreted in the feces (1), the majority are excreted via the kidneys as a glucuronide (1, 9, 13, 14). The elimination half-life is approximately 18 h but can range between 8 and 37 h (13, 14, 17). The LD50 is 338 and 425 mg/kg in mice and rats, respectively (13). The logP, or measure of lipophilicity, has been reported as 4.8 (18) and 5.2 (19).

Treatment of cyclobenzaprine overdose has relied on the removal of toxins from the gastrointestinal tract, as cyclobenzaprine may cause delayed gastric emptying and allow for further absorption (4, 8, 13), and supportive care of clinical signs. Induction of emesis is discouraged in human patients due to alterations in mentation. Activated charcoal may be administered to help reduce enterohepatic recirculation (7). In one case study of a severely affected human, total plasmapheresis was successfully performed to decrease time to clinical resolution (17). Sodium bicarbonate has been used in humans with QRS prolongation (13), and physostigmine has been used in cases of anticholinergic signs refractory to supportive care (8, 11, 13). Additionally, cyproheptadine was used in a case of escitalopram and cyclobenzaprine overdose in which clinical signs resolved after discontinuing the medications (15).

Accidental ingestion of cyclobenzaprine in animals has not been well documented. To date, there are no known publications on cyclobenzaprine ingestion in veterinary medicine. This case series describes the clinical signs and successful treatment with intralipid emulsion (ILE) of two dogs after ingestion of cyclobenzaprine.

## Case summaries

### Case 1

A 6.26-kg 4-year-old female spayed Rat Terrier presented after ingesting between 60 and 160 mg (9.7–25.9 mg/kg) of cyclobenzaprine (10 mg tablets). The ingestion occurred within a 4-h time window, and the dog was brought to the emergency department an hour after discovering the bottle had been chewed. The dog was noted to be growling in the car prior to arrival, which was not its normal behavior.

On presentation to the emergency department (ED), the dog had sinus tachycardia with a persistent heart rate (HR) of 180 beats per minute, a temperature of 38.4˚C (101.1F), and was panting. Agitation, trembling, and vocalizing were also noted. The remainder of the physical exam was unremarkable. The systolic blood pressure was 230 mmHg (reference range 90–140 mmHg) measured via Doppler. A 22 g IV catheter was placed in the right cephalic vein. Point-of-care blood work revealed a packed cell volume (PCV) of 50% (reference range 35–55%) and total solid (TS) of 6 g/dL (6–8.5 g/dL) (60 g/L, 60–85 g/L). The venous blood gas (VBG) (Table 1) was unremarkable other than a mild hyperlactatemia of 2.9 mmol/L (reference range 0–2.5 mmol/L) (26.1 mg/dL, reference range 0–22.5 mg/dL). A complete blood count (CBC) and serum biochemistry evaluation were not performed due to financial limitations. Continuous telemetry was initiated, which revealed persistent sinus tachycardia ranging from 180 to 280 beats per minute (bpm). A urine specific gravity (USG) was concentrated at 1.041 (reference range 1.020–1.040).

**Table 1:** Venous blood gas data collected during hospitalization for each case.

Venous blood gas	Case 1			Case 2	Reference range	Triage (3 PM)	9 PM	6 AM	Triage
**pH**	Error	7.38	7.38	7.41	7.35–7.45
**pCO2 (mmHg)**	40.3	Error	38.4	42.7	0–50.1
**HCT (%)**	53.1	54.6	49.1	45.8	35–55
**K (mEq/L)**	4.7	3.7	4.1	4.2	3.4–5.0
**Na (mmol/L)**	152	148	142	149	140–155
**Ca (mmol/L)**	1.3	1.22	1.26	1.3	1.10–1.40
**Cl (mmol/L)**	120	115	112	112	112–122
**Glu (mg/dL)**	83	97	98	114	60–120
**Lac (mmol/L)**	2.9	2.4	1.8	1.4	0–0.2
**Creatinine (mg/dL)**	0.93	0.89	0.97	0.4	0.6–1.5
**Bicarb (mEq/L)**	Error	Error	21.8	26.3	14–24
**BE (ecf)**	Error	Error	−2.2	2.7	−2–2

The dog was given a 2 mg/kg IV bolus of intralipid^a^ over 15 min and was maintained on a 0.25 mL/kg/min constant rate infusion (CRI) over 1 h. Other therapeutic interventions included IV fluid therapy at 2 mL/kg/h of lactated Ringer’s solution^b^, cyproheptadine^c^ (1.1 mg/kg PO q6h), charcoal with kaolin and sorbitol^d^ (5.5 g/kg once), and charcoal with kaolin^e^ (5.5 g/kg q8h for three additional doses). Serum and urine samples were collected 30 min prior to intralipid administration and 1 h after completion. Cyclobenzaprine levels were measured via high-performance liquid chromatography/tandem mass spectrometry at NMS Labs, Horsham, PA. The initial serum level of cyclobenzaprine was 99 ng/mL, and the post-ILE serum level was 100 ng/mL (reference range 4–40 ng/mL) (Table 2). The initial urine concentration of cyclobenzaprine was over 400 ng/mL, and the post-ILE concentration was 97 ng/mL (no reference range) (Table 2).

**Table 2:** Pre-ILE and Post-ILE serum and urine levels of cyclobenzaprine from case 1.

	Case 1
**Pre-serum (ng/ml)**	99
**Post-serum (ng/ml)**	100
**Pre-urine (ng/ml)**	>400
**Post-urine (ng/ml)**	97

Pre-serum and urine samples were collected within 30 min of ILE administration, and post-serum and urine samples were collected within an hour of completing the ILE CRI.

Immediately after the ILE infusion, the tachycardia resolved, and the HR remained between 90 and 130 bpm. Blood pressure was 118 mmHg (#3 right hind limb) (reference range 90–140 mmHg). The agitation and trembling also appeared to resolve after the intralipid therapy. The USG decreased to 1.025 (reference range 1.020–1.040) after 6 h on IV LRS at 2 mL/kg/h. A recheck of PCV and TS showed 46% (reference range 35–55%) and 5.4 g/dL (reference range 6–8.5 g/dL) (54 g/L, reference range 60–85 g/L). Repeat VBG was unremarkable (Table 1).

The dog had a great appetite in the hospital and was discharged the day after the presentation with no additional medications. Mild diarrhea reportedly developed at home and resolved over the next 24 h without intervention. The dog was reportedly doing well at home.

### Case 2

A 2.5-kg 5-month-old female intact mixed-breed dog presented for the ingestion of an unknown number of 5 mg cyclobenzaprine tablets 2–3 h prior to presentation. The dog appeared dull and uncoordinated at home and began drooling. One episode of urinary incontinence was reported.

On presentation to the ED, the dog had a mildly elevated rectal temperature of 39.3˚C (102.8F), tachycardia of 168 bpm, quiet mentation, spinal ataxia in all four limbs, and an absent gag reflex. Blood pressure was not obtained on intake.

The initial PCV and TS were 43% (reference range 35–55%) and 5.6 g/dL (reference range 6–8.5 g/dL) (56 g/L, reference range 60–85 g/L) with a normal VBG (Table 1). A 22 g IV catheter was placed in the left cephalic vein. Due to the lack of gag reflex, the decision was made to intubate and perform orogastric decontamination via gastric lavage. Propofol^f^ (0.6 mg/kg IV) was administered to facilitate rapid intubation, and the dog was anesthetized. The dog was extubated and reintubated with a smaller, 4.5 French endotracheal tube. The patient was placed in sternal recumbency. Gastric lavage was successfully performed by infusing 60 mL of warm tap water through the tube and emptying by gravity into an empty bucket. This was repeated seven times until recovery of clear gavage fluid was noted. It was not recorded if intact pills were obtained. The larynx and oral cavity were then suctioned, and 7 g/kg of charcoal and kaolin with sorbitol^d^ was administered via the orogastric tube prior to its removal.

Upon recovery, the gag reflex was assessed to be present, there was mild subcutaneous emphysema palpated around the neck, and there was concern for an iatrogenic tracheal tear from intubation. Thoracic radiographs showed moderate intermittent esophageal dilation, moderate gastric undigested material with gastric pneumatosis, and scant cervical subcutaneous emphysema. The subcutaneous emphysema resolved without further treatment.

The dog was treated with a 1.5 mL/kg bolus of ILE^a^ and a 0.25 mL/kg/min continuous rate infusion over 1 h, IV fluid therapy of 2.5 mL/kg/h of lactated Ringer’s solution^b^, maropitant^g^ (1 mg/kg IV q24h), and pantoprazole^h^ (1 mg/kg IV q12h). A CBC and a serum biochemistry profile were not performed due to financial constraints; therefore, samples were not obtained for measurement of cyclobenzaprine levels.

Following gastric lavage and ILE administration, the tachycardia resolved, and the HR remained between 85 and 125 BPM with a normal sinus rhythm monitored via continuous ECG for the remainder of hospitalization. The gag reflex remained intact throughout hospitalization, and the dog ate well with no observable complications. The dog was discharged 1 day later with no further medications. The dog was doing well at home several weeks after discharge.

## Discussion

This is the first case series in dogs treated with ILE following cyclobenzaprine ingestion. Clinical signs were similar to those reported in people, with mentation and cardiovascular changes noted in each case. The dog in case 1 demonstrated abnormal behavior (growling in the car, agitation, tremors, and vocalization), tachycardia, and hypertension. The dog from case 2 was more severely affected by dull mentation, incoordination, hypersalivation, tachycardia, hypertension, an absent gag reflex, and mild hyperthermia. In veterinary medicine, it can sometimes be difficult to determine if agitation, tachycardia, and hypertension are pathologic or driven by anxiety, or “white coat syndrome.” In case 1, tachycardia was monitored with continuous telemetry with no handling or restraint necessary for repeat assessment, and the tachycardia resolved after ILE even though the dog’s surroundings had not changed. Blood pressure should be monitored closely given that both hypertension and hypotension, though less common, are reported in human literature and may require vasopressor therapy for cardiovascular support (3).

Emesis was not induced in case 1 given the time lapse between potential ingestion and presentation. Case 2 underwent gastric lavage given the absent gag reflex. However, it is reasonable to consider gastric lavage in cases with moderate-to-severe clinical signs depending on the dose ingested and the neurologic status of the patient. Complete gastric emptying in normal dogs is about 5–10 h given a full meal (8 g/kg and an additional 5–7 mL/kg of contrast); however, dogs fed a smaller amount had faster gastric emptying times (20). Gastric lavage may be advantageous in cases where induction of emesis cannot be performed when ingestion is within 10 h of presentation. Both cases received activated charcoal to adsorb any remaining toxin and reduce enterohepatic recirculation. Cyproheptadine was administered in case 1 due to its mechanism of action as a serotonin antagonist, and there was an initial concern for serotonin syndrome. Cyproheptadine does not seem to be standard in treating cyclobenzaprine toxicity in humans, with one report of its use in a case with concurrent escitalopram ingestion. There is a concern that cyproheptadine is also bound by activated charcoal and may be ineffective (21). However, care was taken to stagger administration with charcoal to avoid altered absorption in case 1.

ILE is a relatively new and increasingly popular treatment for various toxicoses in veterinary medicine, though treatment guidelines and safety information are lacking. ILE is mostly used in the treatment of toxicity due to fat-soluble substances, and many reports exist of its utility in veterinary patients (16, 18, 22–24).

ILE was initially developed for treating systemic toxicity due to local anesthetic drugs and has since been implemented for the treatment of antiarrhythmic, psychotropic, and antimalarial drug overdoses along with organophosphate exposure (22–25). Highly lipid-soluble drugs will have a logP, a measure of lipophilicity, greater than 2 (18). The logP of cyclobenzaprine has been reported as 4.8 (18) and 5.2 (19), making ILE a reasonable treatment option for animals that have ingested fat-soluble substances. ILE is made up of sterile, nanometer-sized droplets of triglyceride oils in water that are stabilized by phospholipid surfactants (16, 22). The particle size of the triglyceride droplets ranges between 200 and 600 nm, similar in size to a chylomicron (20). The osmolality of ILE is 270 mOsm/L, and it has a zeta potential of −45 to −40 mV, making the particle negatively charged (22).

The mechanism of how ILE clears toxins from the body is not well understood. Many different theories include the lipid sink, lipid shuttle, and fatty acid uptake into the mitochondria, activation of a cytoprotective cascade, inhibition of nitric oxide, and promotion of calcium entry into the cells (16, 18, 22–26). The lipid sink theory is the most understood. The lipids compartmentalize the offending toxin into a lipid phase within the vasculature; therefore, the toxin cannot bind to the target receptors in the tissues (16, 18, 23–26). A new equilibrium between the vasculature, tissue, and plasma is formed, and there is less toxin present to cause systemic effects. The toxin may also bind to the negatively charged exterior of the lipid spherule for further clearance (20). The lipid shuttle theory promotes the removal of toxin from the peripheral tissue into the lipid emulsion and then shuttles it to the liver for metabolism (16, 22, 23). This theory hypothesizes that the toxin will be cleared faster within the lipid emulsion than it would on its own.

When the toxin is suspected to be compartmentalized by the lipid in the lipid phase within the vascular space, the concentration of the toxin is temporarily increased. This may explain why the plasma concentrations post-ILE increased in the first case. Akyol and Gokbulut (27) documented this finding in rabbits that were given ivermectin and carprofen, and plasma concentrations were measured in those that did not receive ILE and those that did. Those that received ILE had greater plasma concentrations of ivermectin and carprofen while also having a smaller volume of distribution (27). These findings support the lipid sink theory that ILE will draw the toxin out of the tissues and into the vascular space for excretion (27). This phenomenon has also been noted in a case study by Clark et al. (25), in which ILE was used to treat ivermectin toxicosis in a Border Collie. After the initial ILE bolus, ivermectin levels increased within the plasma and then subsequently decreased (25). Additionally, this phenomenon has been noted in bupropion and lamotrigine overdose in a man (26).

The decrease in the urine cyclobenzaprine levels in case 1 may be due to excretion through other routes, such as the gastrointestinal tract. There is rationale for this theory, given enterohepatic recirculation is known to occur with this drug, and the biliary tract empties the drug metabolites back into the upper gastrointestinal tract in a cyclic fashion until excretion occurs via the feces.

While ILE is generally safe to use, there have been adverse effects in both human and veterinary medicine. Hyperlipidemia will occur after use and has been reported to last for up to 48 h (16). Hyperlipidemia can cause corneal lipidosis and pancreatitis and may interfere with laboratory testing of electrolytes, hematocrit, liver function tests, and coagulation factors (16, 18). In humans, ILE has been implicated in acute lung injury, acute kidney injury, acute respiratory distress syndrome, cardiac embolism, fat overload syndrome, and increased susceptibility to infection (16, 18). Additionally, ILE may increase the gastrointestinal absorption of fat-soluble drugs and worsen clinical signs of toxicity (22).

Limitations of this study include the fact that it is retrospective in nature, and one case did not have pre-ILE and post-ILE serum or urine samples collected. Complete blood counts and serum biochemistry profiles were not performed in either case to detect any changes induced by cyclobenzaprine on the hemogram or biochemistry panel. However, this is not an expected finding based on data in human patients.

This case series is the first to document clinical signs after cyclobenzaprine ingestion in two dogs. Tachycardia, anxiety, altered mentation, and hypertension were common findings in both dogs, and response to ILE and supportive care was excellent. ILE may be a valid treatment option in cases of cyclobenzaprine overdose. However, future studies may assess the time to resolution of clinical signs without ILE treatment.

### Manufacturer information

- Intralipids—B Braun Medical, Inc., Bethlehem, PA
- Lactated Ringer’s solution—Dechra Veterinary Products, Overland Park, TX
- Cyproheptadine oral suspension—Compounded at UFVH Pharmacy, Gainesville, FL
- Charcoal with kaolin and sorbitol—Lloyd, Inc., Shenandoah, IA
- Charcoal with kaolin—Lloyd, Inc., Shenandoah, IA
- Propofol—Zoetis, Inc., Kalamazoo, MI
- Maropitant injectable—Zoetis, Inc., Kalamazoo, MI
- Pantoprazole—AuroMedics Pharma, LLC East Windsor, NJ

## Data availability statement

The original contributions presented in the study are included in the article/Supplementary material, further inquiries can be directed to the corresponding author.

## Ethics statement

The requirement of ethical approval was waived by University of Florida Institutional Animal Care and Use Committee for the studies involving animals because data was collected from animal records that were previously treated within the hospital. The studies were conducted in accordance with the local legislation and institutional requirements. Written informed consent was obtained from the owners for the participation of their animals in this study.

## Author contributions

KD: Investigation, Writing – original draft. AO: Conceptualization, Resources, Supervision, Writing – review & editing. MB: Conceptualization, Writing – review & editing.
