# Supplementary material for: Case report: Treatment of cyclobenzaprine ingestion in two dogs with intravenous intralipid therapy
Source: Front Vet Sci. 2024 Feb 12;11:1354028. doi: 10.3389/fvets.2024.1354028 (PMC10894977; doi:10.3389/fvets.2024.1354028)
Supplement: Supplementary file 1 [file Data_Sheet_1.PDF]

CARE case report guidelines

CARE Checklist of information to include when writing a case report

(cc) BY-NC-ND

Topic	Item	Checklist item description	Reported on Line
Title	1	The diagnosis or intervention of primary focus followed by the words "case report"	1-2
Key Words	2	2 to 5 key words that identify diagnoses or interventions in this case report, including "case report"	20
Abstract (no references)	3a	Introduction: What is unique about this case and what does it add to the scientific literature?	4-5
	3b	Main symptoms and/or important clinical findings	7-11, 12-15
	3c	The main diagnoses, therapeutic interventions, and outcomes	11, 12, 15-17
	3d	Conclusion—What is the main "take-away" lesson(s) from this case?	18-19
Introduction	4	One or two paragraphs summarizing why this case is unique (**may include references**)	29 -39, 65 - 69
Patient Information	5a	De-identified patient specific information.	71, 107
	5b	Primary concerns and symptoms of the patient.	74 -75, 109 - 110
	5c	Medical, family, and psycho-social history including relevant genetic information	N/A
	5d	Relevant past interventions with outcomes	55 - 64
Clinical Findings	6	Describe significant physical examination (PE) and important clinical findings.	76 - 80, 111 -113
Timeline	7	Historical and current information from this episode of care organized as a timeline	N/A
Diagnostic Assessment	8a	Diagnostic testing (such as PE, laboratory testing, imaging, surveys).	80 -87, 99 - 102, 111 - 117,
	8b	Diagnostic challenges (such as access to testing, financial, or cultural)	85, 132 - 134
	8c	Diagnosis (including other diagnoses considered)	72, 108
	8d	Prognosis (such as staging in oncology) where applicable	N/A
Therapeutic Intervention	9a	Types of therapeutic intervention (such as pharmacologic, surgical, preventive, self-care)	88 - 92, 119 - 125, 130 - 132
	9b	Administration of therapeutic intervention (such as dosage, strength, duration)	88 - 92, 130 - 132
	9c	Changes in therapeutic intervention (with rationale)	N/A
Follow-up and Outcomes	10a	Clinician and patient-assessed outcomes (if available)	103 - 105, 138 - 139
	10b	Important follow-up diagnostic and other test results	N/A
	10c	Intervention adherence and tolerability (How was this assessed?)	N/A
	10d	Adverse and unanticipated events	N/A
Discussion	11a	A scientific discussion of the strengths AND limitations associated with this case report	211 -215
	11b	Discussion of the relevant medical literature **with references**.	22 - 62, 167 - 210
	11c	The scientific rationale for any conclusions (including assessment of possible causes)	141 - 166
	11d	The primary "take-away" lessons of this case report (without references) in a one paragraph conclusion	210 - 220
Patient Perspective	12	The patient should share their perspective in one to two paragraphs on the treatment(s) they received	N/A
Informed Consent	13	Did the patient give informed consent? Please provide if requested	Yes ☒ No ☐
